# Phylogeny of the plant 4/1 proteins

**DOI:** 10.1016/j.dib.2015.11.041

**Published:** 2015-11-25

**Authors:** Sergey Y. Morozov, Andrey G. Solovyev, Alexey V. Troitsky

**Affiliations:** A. N. Belozersky Institute of Physico-Chemical Biology, Lomonosov Moscow State University, 119992 Moscow, Russia

## Abstract

The Nt-4/1 protein of unknown function has been shown to be alpha-helical and predominantly expressed in conductive tissues of tobacco plants. So far, obvious Nt-4/1 orthologs were found only in flowering plants. We report the analysis of 4/1 genes and the encoded proteins of lower land plants (Morozov et al., 2015) [1]. In this data article, we present two phylogenetic trees of angiosperm 4/1 proteins together with orthologs from liverworts, lycophytes, ferns and gymnosperms.

**Specifications Table**

**Table:** 

Subject area	*Biology*
More specific subject area	*Phylogenetics*
Type of data	*Figure (Phylogenetic trees)*
How data was acquired	*Phylogenies were acquired using Fast Minimum Evolution and Neighbour-joining methods at NCBI (COBALT) and TREECON packages*
Data format	*Analyzed*
Experimental factors	*Amino acid sequences were retrieved from NCBI and/or 1KP databases (see below)*
Experimental features	*Sequences were aligned using NCBI protein Multiple Alignment Tool (see below)*
Data source location	*NCBI:*http://www.ncbi.nlm.nih.gov/
*TREECON:*http://bioinformatics.psb.ugent.be/downloads/psb/Userman/treeconw.html
*1KP:*http://www.onekp.com
Data accessibility	*With this article*

**Value of the data**

• The 4/1 genes are low-copy genes and its molecular evolution is intriguing to understand their phylogenies among plants.
• Data on phylogenies separately estimated using Fast Minimum Evolution and neighbour-joining methods enable researchers to examine how the topologies differ from each other.
• Data on phylogenies of 4/1 proteins is intriguing to understand their unique features.
• Data on phylogenies of 4/1 proteins enable researchers to infer the possible ranges of time frames in the divergence events of 4/1 low-copy genes and its molecular evolution in general.

## 1 Data, experimental design, materials and methods

**Fig. 1:**
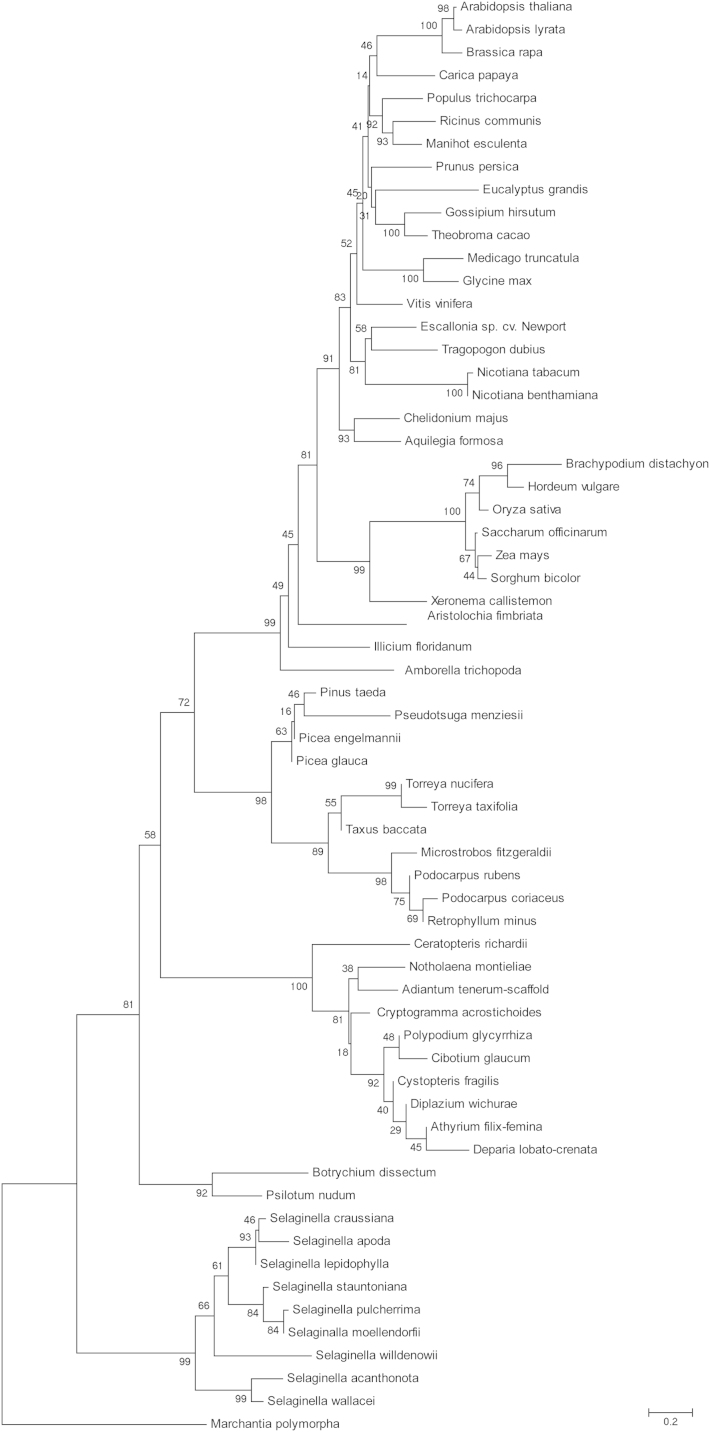
Neighbour-joining (NJ) tree based on the 4/1 protein sequences showing the phylogenetic relationship between 4/1 polypeptides from land plants. The numbers indicate the NJ bootstrap values for 1000 replicates.

**Fig. 2:**
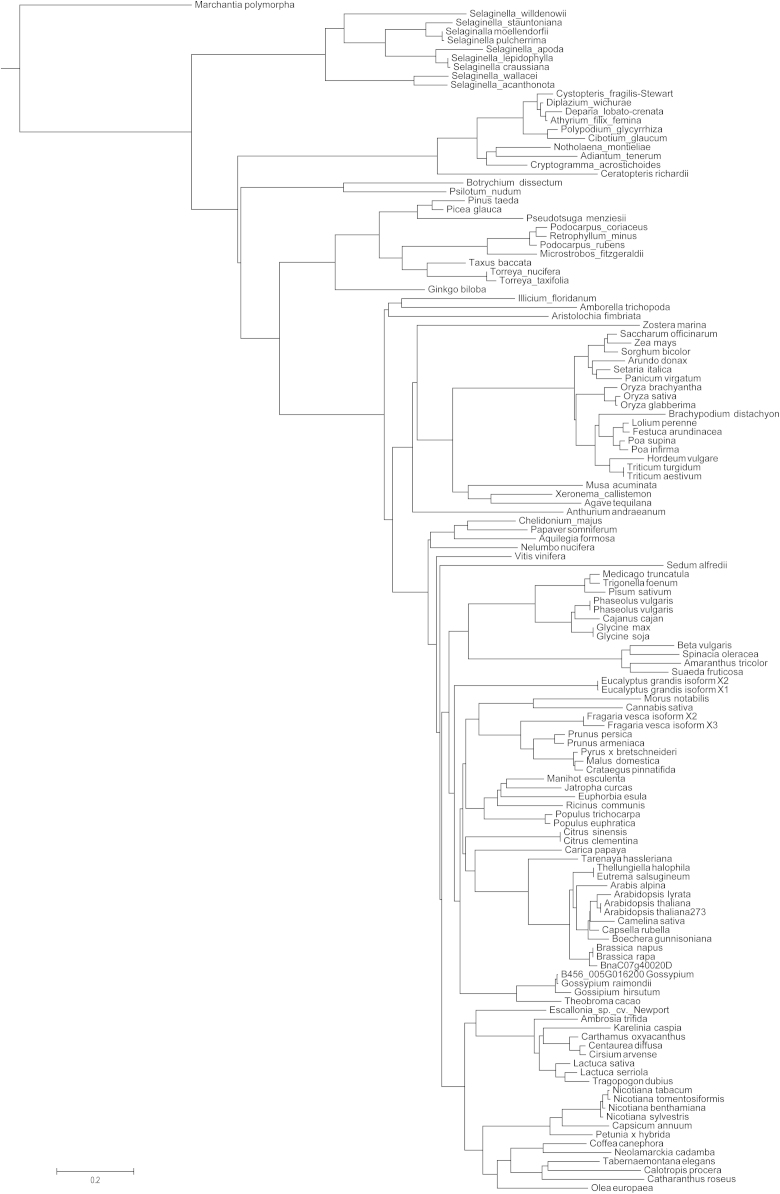
The phylogenetic tree based on analysis of the 134 aligned 4/1 proteins from land plants. Fast Minimum Evolution tree was obtained at http://www.ncbi.nlm.nih.gov/tools/cobalt/ with the use of default parameters.

The phylogenetic tree obtained using COBALT (http://www.ncbi.nlm.nih.gov/tools/cobalt/) Fast Minimum Evolution method for 4/1 proteins from 62 plant species was presented in [1]. The data shown here represent the phylogenetic tree of 62 sequences of 4/1 proteins separately reconstructed using a neighbour-joining method by TREECON 1.3b package (Fig. 1) and combined phylogenetic tree for 134 plant 4/1 proteins constructed by a COBALT Fast Minimum Evolution method (Fig. 2). All the sequence data used in this data article were retrieved from NCBI (http://www.ncbi.nlm.nih.gov/) and 1KP databases (http:// www.onekp.com). These sequences were aligned by NCBI protein Multiple Alignment Tool software using default parameters.
